# Building a RAFFT: Impact of a professional development program for women faculty and residents in emergency medicine

**DOI:** 10.1002/aet2.10763

**Published:** 2022-06-23

**Authors:** Simiao Li‐Sauerwine, Kimberly Bambach, Jillian McGrath, Jennifer Yee, Creagh T. Boulger, Katherine M. Hunold, Jennifer Mitzman

**Affiliations:** ^1^ Department of Emergency Medicine The Ohio State University Columbus Ohio USA; ^2^ Nationwide Children’s Hospital Columbus Ohio USA

## Abstract

**Background:**

Women comprise 28% of faculty in academic departments of emergency medicine (EM) and 11% of academic chairs. Professional development programs for women are key to career success and to prevent pipeline attrition. Within emergency medicine, there is a paucity of outcomes‐level data for such programs.

**Objectives:**

We aim to measure the impact of a novel structured professional development curriculum and mentorship group (Resident and Faculty Female Tribe, or RAFFT) within an academic department of EM.

**Methods:**

This prospective single‐center curriculum implementation and evaluation was conducted in the academic year 2020–2021. A planning group identified potential curricular topics using an iterative Delphi process. We developed a 10‐session longitudinal curriculum; a postcurriculum survey was conducted to assess the perceived benefit of the program in four domains.

**Results:**

A total of 76% of 51 eligible women attended at least one session; for this project we analyzed the 24 participants (47%) who attended at least one session and completed both the pre‐ and the postsurvey. The majority of participants reported a positive benefit, which aligned with their expectations in the following areas: professional development (79.2%), job satisfaction (83.3%), professional well‐being (70.8%), and personal well‐being (79.2%). Resident physicians more often reported less benefit than expected compared to fellow/faculty physicians. Median perceived impact on career choice and trajectory was positive for all respondents.

**Conclusions:**

Success of this professional development program was measured through a perceived benefit aligning with participant expectations, a positive impact on career choice and career trajectory for participants in each career stage, and a high level of engagement in this voluntary program. Recommendations for the successful implementation of professional development programs include early engagement of stakeholders, the application of data from a program‐specific needs assessment, early dissemination of session dates to allow for protected time off, and structured discussions with appropriate identification of presession resources.

## INTRODUCTION

Gender inequity in medical school admissions has improved over the past decade. In recent years, the proportion of female graduates of both Liaison Committee on Medical Education– and osteopathic‐accredited U.S. medical schools is now just above 50%.^1^, ^2^ Despite this increased representation, the proportion of women in emergency medicine (EM) residency programs has remained about 36%. Representation in leadership positions in EM is even lower, with women comprising 28% of faculty in academic departments of EM and 11% of academic EM chairs.^3^, ^4^

A study from 2020 based on data from the Association of American Medical Colleges demonstrated that over a 35‐year period, women physicians in academic medical centers were less likely than men to be promoted to the rank of associate professor, full professor, or department chair with no apparent narrowing of this gap over time.^5^, ^6^, ^7^, ^8^ Women in academic EM are paid significantly less, but work more clinical hours than their male colleagues^9^, ^10^ and are more prone to gender discrimination.^11^ Women trainees in EM are even more vulnerable to bias, with robust data reflecting differences in selection and matriculation,^12^, ^13^, ^14^, ^15^ faculty and nursing evaluation of performance,^16^, ^17^, ^18^ patient perception,^19^ and attrition.^20^

In light of such data, there is a growing recognition of the need for support for women in academic medicine. Professional development programs for faculty are key to career satisfaction and success and to prevent pipeline attrition.^21^, ^22^, ^23^ It is important that these programs are tailored to fit the specific needs of women physicians.^20^, ^24^, ^25^, ^26^, ^27^ Some professional development and mentorship programs designed to support women in academic medicine and EM have been described in the literature.^21^, ^22^, ^23^, ^28^, ^29^, ^30^, ^31^, ^32^ While these programs are valuable and offer pragmatic solutions for supporting women physicians, there is a paucity of quantitative data on the impact of these programs on participants’ professional trajectories and personal and professional well‐being.

In the 2020–2021 academic year, we implemented a professional development curriculum and mentorship group for women faculty and residents (Resident And Faculty Female Tribe, or RAFFT), in the department of EM at our institution. In this study, we present a framework for desired professional development topics for women‐identifying faculty and residents. We further report the impact of this longitudinal monthly curriculum on specific measures of professional development, success, and satisfaction.

## METHODS

### Study design and setting

This prospective single‐center curriculum design, implementation, and evaluation was conducted during a single academic year, from July 2020 to June 2021. This study was deemed exempt by the institutional review board at our institution.

### Study population and protocol

A self‐selected planning group of five faculty and four chief residents in EM interested in addressing professional development for women‐identifying faculty and residents convened to plan a 1‐year curriculum including determination of topics and optimal program structure. All members of the planning group were women‐identifying; one member was underrepresented in medicine (URiM) and three others were from non‐URiM historically excluded groups. The faculty group included three associate and two assistant professors. One lead has significant prior work in the area of gender issues in EM, one is nationally recognized for Delphi expertise in curriculum development in EM, and another has substantial Delphi knowledge in the area of simulation‐based medical education. All have expertise in curriculum development. A list of potential curricular topics was created by the planning group using an iterative Delphi process. After initially brainstorming for potential curricular topics, the planning group convened to iteratively refine and rank potential topics by perceived need. These topics were then assessed in a digital preimplementation survey sent to all 51 women faculty and residents on a 5‐point Likert scale for “current knowledge of (topic)” and “desire to learn more about (topic).” Three reminder emails were sent out to this group and all collected data were anonymized. Based on survey data, monthly longitudinal curriculum comprised of 10 sessions was then developed for the months of August 2020–May 2021 (Table 1). Prior to each session, the planning group identified appropriate presession reference materials (e.g., published articles, audio and video resources) for the chosen topic and curated a discussion guide using an iterative consensus building process. Potential resources were initially identified by planning committee members through a search of the literature, social media resources, podcasts, and other publications such as the Harvard Business Review. Prior to each session the planning committee was given an opportunity to comment on and add to the resources selected by the session leads. Curricular sessions were led by faculty and residents and conducted in person or via Zoom as COVID‐related gathering restrictions permitted. At the end of the academic year, a postcurriculum survey was conducted using the same 5‐point Likert scale in the preimplementation survey to assess knowledge acquisition and perceived benefit of the curriculum to respondents with respect to professional development and personal and professional well‐being. Both the pre‐ and the postimplementation surveys were housed on Google Forms (Appendix S1 and S2).

**TABLE 1:** Topics for 10‐session longitudinal women in EM curriculum

Session	Month	Topic
1	August	Supporting each other professionally
2	September	Mentorship—how to seek it out, what to ask for
3	October	Thriving clinically—nursing and staff communication
4	November	Professional advancement—advocating for yourself
5	December	Career exploration and job‐specific mentorship
6	January	Imposter syndrome
7	February	Work–life balance—relationships
8	March	Work–life balance—raising a family
9	April	Professional advancement—salary and contract negotiation
10	May	Pearls of wisdom and senior graduation celebration

### Key outcome measures and data analysis

Attendance is presented stratified by junior residents (PGY‐1/2), senior residents (PGY‐3+), and fellows/faculty. The pre‐ and postcurriculum deidentified survey data are compared descriptively. This study was not meant to test a hypothesis, but rather to describe the program's impact at one institution. Accordingly, no power or sample size analysis was conducted and no formal statistical testing was conducted.

The expected (pre‐) and experienced (post‐) benefits are presented as *n* (%) overall and stratified as follows: those experiencing the same benefit as expected (negative, neutral, or positive) and different benefit than expected (more or less). We provide attendance and level of training for those experiencing a different benefit than expected. The Likert scale responses for impact on career choice and trajectory are presented as boxplots for junior residents (PGY‐1/2), senior residents (PGY‐3+), and fellows/faculty.

## RESULTS

**FIGURE 1:**
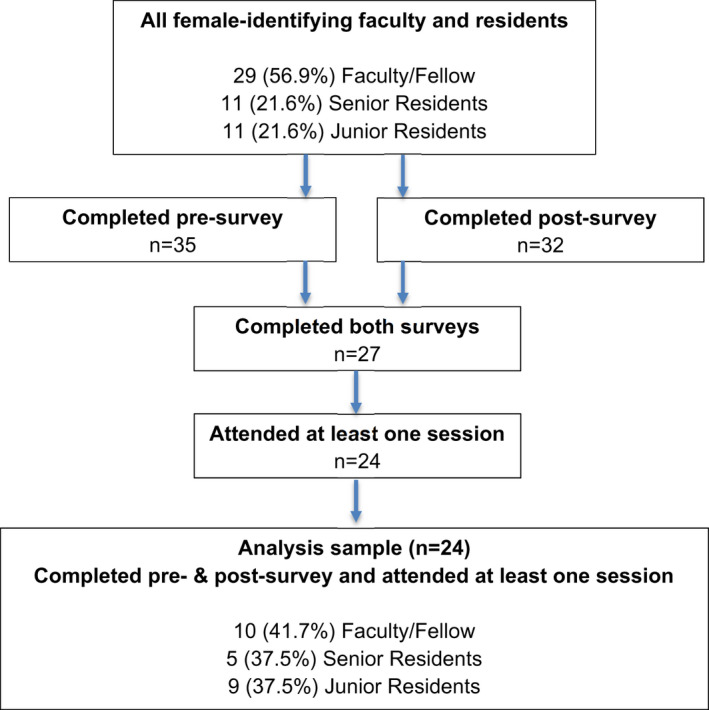
Flow diagram.

A total of 51 women‐identifying faculty, fellows, and residents were eligible for inclusion (22 faculty/fellows and 18 residents in academic year 2020–2021). Thirty‐nine women (76%) attended at least one session. The presurvey was completed by 35 participants and the postsurvey by 32; 27 participants responded to both. To be eligible for the analysis sample, participants had to have attended at least one session and completed both the pre‐ and the postsurveys, leaving 24 (47% of total eligible participants) for analysis (Figure 1).

**FIGURE 2:**
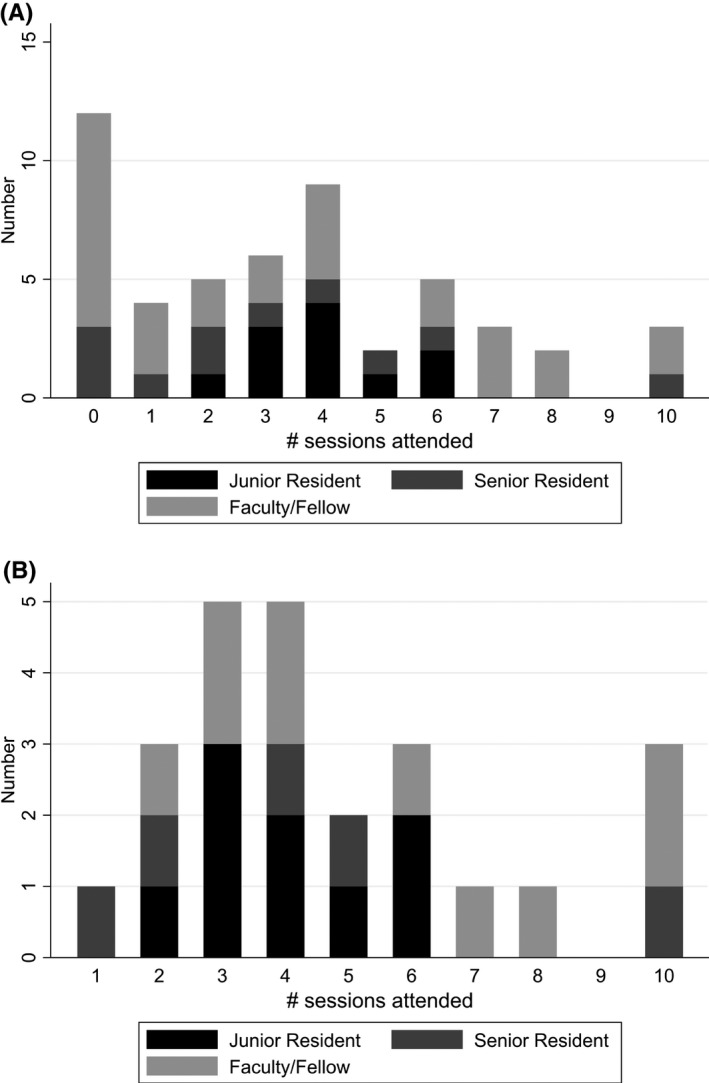
(A) Attendance of RAFFT sessions by faculty, senior residents, and junior residents (*n* = 51). (B) Attendance of RAFFT sessions by faculty, senior residents, and junior residents included in analysis (*n* = 24). RAFFT, Resident And Faculty Female Tribe.

The distribution of attendance for all women‐identifying faculty, fellows, and residents is shown in Figure 2A. Attendance in the analysis sample is shown in Figure 2B. Of those included in the analysis, the majority attended three or four sessions. One senior resident and four fellow/faculty members attended seven or more sessions.

**FIGURE 3:**
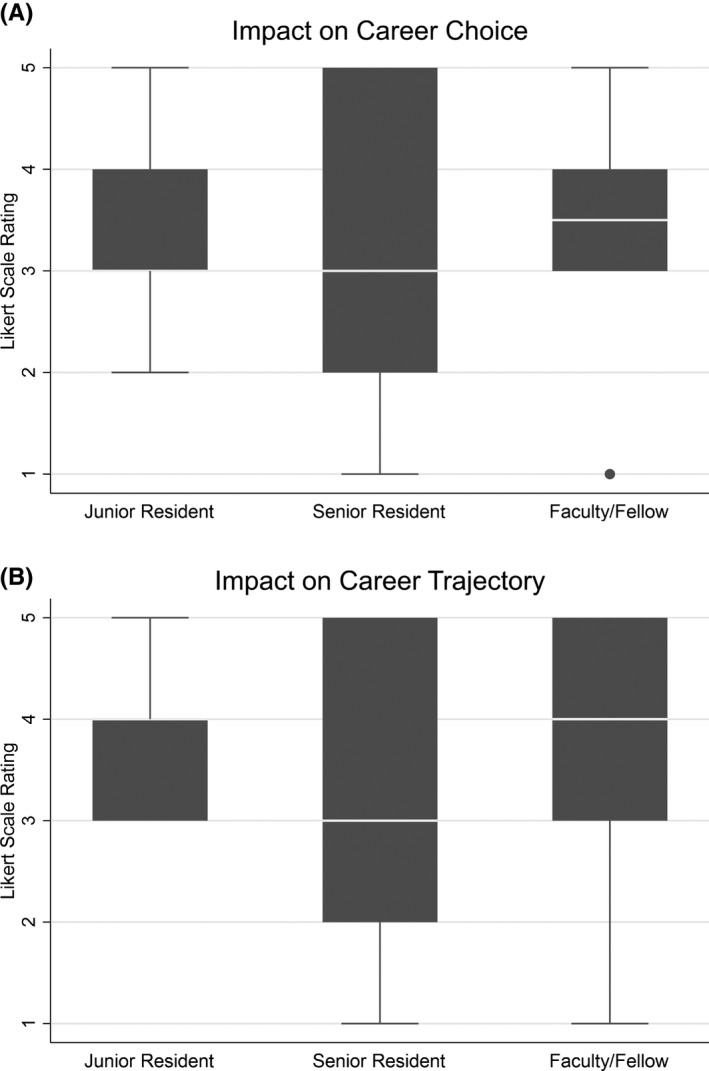
Impact of RAFFT on career choice and career trajectory stratified by junior resident (*n* = 9), senior resident (*n* = 5), and faculty/fellow (*n* = 10) RAFFT, Resident And Faculty Female Tribe.

The majority of participants reported a positive benefit, which aligned with their expectations in the following areas: professional development (79.2%), job satisfaction (83.3%), professional well‐being (70.8%), and personal well‐being (79.2%). A minority of participants reported an impact opposite their initial expectations, with some reporting less benefit than anticipated in professional development (12.5%), job satisfaction (12.5%), professional well‐being (25.0%), and personal well‐being (12.5%). Two participants (8.3%) reported a positive benefit despite initial negative expectations in professional development, and one participant (4.2%) reported a positive benefit despite initial negative expectations in job satisfaction, professional well‐being, and personal well‐being (Table 2). Resident physicians more often reported less benefit than expected compared to fellow/faculty physicians (Table 3). Median impact on career choice and trajectory was positive for all respondents; senior resident physicians reported a lower median impact compared to junior resident and faculty/fellow physicians (Figure 3).

**TABLE 2:** Expected and actual benefit of RAFFT in four areas assessed both before and after Year 1 of the program in full sample (*n* = 24)

	Expected and actual benefit					Notes
	Same			Different		
Professional development	19 (79.2)			5 (20.8)		Among those who reported less benefit than expected: one attended only one session, one attended four and one attended six; two were senior residents and one was a fellow/faculty
	Negative	Neutral	Positive	Less	More	
	0 (0.0)	0 (0.0)	19 (79.2)	3 (12.5)	2 (8.3)	
Job satisfaction	20 (83.3)			4 (16.7)		Among those who reported less benefit than expected: one attended only one session, one attended four and one attended six; two were senior residents and one was a fellow/faculty
	Negative	Neutral	Positive	Less	More	
	0 (0.0)	0 (0.0)	20 (83.3)	3 (12.5)	1 (4.2)	
Professional well‐being	17 (70.8)			7 (29.2)		Among those who reported less benefit than expected: one attended only one session, one attended two, one attended three, one attended four, and one attended six; two were junior residents, two were senior residents, and two were fellows/faculty
	Negative	Neutral	Positive	Less	More	
	0 (0.0)	0 (0.0)	17 (70.8)	6 (25.0)	1 (4.2)	
Personal well‐being	20 (83.3)			4 (16.7)		Among those who reported less benefit than expected: one attended only one session, one attended three and one attended four; one was a junior resident and two were senior residents
	Negative	Neutral	Positive	Less	More	
	0 (0.0)	1 (4.2)	19 (79.2)	3 (12.5)	1 (4.2)	

Note

All data are presented as *n* (%).

Abbreviations: RAFFT, Resident And Faculty Female Tribe.

**TABLE 3:** Expected and actual benefit of RAFFT in four areas assessed both before and after Year 1 of the program stratified by faculty/fellow (*n* = 10) and resident (*n* = 14)

	Expected and actual benefit									
	Faculty/fellow					Resident				
	Same			Different		Same			Different	
Professional development	8 (80.0)			2 (20.0)		11 (78.6)			3 (21.4)	
	Negative	Neutral	Positive	Less	More	Negative	Neutral	Positive	Less	More
	0 (0.0)	0 (0.0)	8 (80.0)	1 (10)	1 (10)	0 (0.0)	0 (0.0)	11 (78.6)	2 (14.3)	1 (7.1)
Job satisfaction	9 (90.0)			1 (10.0)		11 (78.6)			3 (21.4)	
	Negative	Neutral	Positive	Less	More	Negative	Neutral	Positive	Less	More
	0 (0.0)	0 (0.0)	9 (90.0)	1 (10.0)	0 (0.0)	0 (0.0)	0 (0.0)	11 (78.6)	2 (14.3)	1 (7.1)
Professional well‐being	8 (80.0)			2 (20.0)		9 (64.3)			5 (35.7)	
	Negative	Neutral	Positive	Less	More	Negative	Neutral	Positive	Less	More
	0 (0.0)	0 (0.0)	8 (80.0)	2 (20)	0 (0.0)	0 (0.0)	0 (0.0)	9 (64.3)	5 (38.6)	1 (7.1)
Personal well‐being	10 (100.0)			0 (0.0)		10 (71.4)			4 (28.6)	
	Negative	Neutral	Positive	Less	More	Negative	Neutral	Positive	Less	More
	0 (0.0)	0 (0.0)	10 (100.0)	0 (0.0)	0 (0.0)	0 (0.0)	1 (7.1)	9 (64.3)	3 (21.4)	1 (7.1)

Note

All data are presented as *n* (%).

Abbreviations: RAFFT, Resident And Faculty Female Tribe.

## DISCUSSION

We demonstrate the successful conception and implementation of a women in EM professional development program. Expectations for RAFFT impact on professional development, job satisfaction, professional well‐being, and personal well‐being were met for a majority of participants, regardless of academic rank, in the inaugural year of this program. We demonstrated a modest positive impact of the program on career choice and career trajectory in all categories of participants. Not all participants completed the pre‐ and postsurveys, which limits a complete understanding of program impact. However, attendance was robust with 76% of women attending at least one session and an average attendance per session of 34% of women physicians in our department. This is a marker of success when considering busy resident, fellow, and faculty schedules with various competing obligations.

While the majority of residents expected and perceived a positive benefit, several reported less benefit than expected. Attendance of those reporting less benefit than expected was low to average. Discrepancy between expected and perceived benefit was most pronounced in the domain of professional well‐being. We hypothesize that increased professional stressors related to the COVID‐19 pandemic may have been a contributing factor. Fellows and faculty reported more benefit in the postsurvey than expected prior to program participation, which may reflect a bridged gap in midcareer faculty development within our department.^31^, ^33^ Senior residents reported a smaller impact on career choice and career trajectory, which may reflect the specific timing of this program in the context of a job search within their final year of training.

This novel curriculum builds on prior published professional development curricula for women in EM in several ways. Although various longitudinal curricula have been described, our group utilized a flipped‐classroom model with several curated resources that addressed a specific theme for each session.^34^ This differs from other programs described and previous iterations of programming for women in our own department that approached professional development with less content specificity.^22^, ^28^, ^29^, ^30^, ^31^ Discussion during sessions focused on the chosen topic and was led by RAFFT planning group members who would volunteer to lead per topic interest and availability. At times, nonplanning group residents with specific interest in a topic led the session in conjunction with planning group members. Encouraging women of all academic ranks to lead sessions promoted engagement and the incorporation of varied perspectives. To promote community and consistency in establishing a new program, session dates for the year were publicized ahead of time allowing for clinical time off requests. Sessions were held on a monthly basis alternating days and evenings to facilitate attendance. This provided women faculty a much‐desired forum to mentor residents with advanced planning and adhering to best practices,^35^, ^36^ as participants had previously found informal and impromptu opportunities (e.g., month‐of scheduling or postshift social events) more difficult to incorporate with coordination of competing work and nonwork demands.

Furthermore, implementation of this program was successful in several less tangible ways. The existence of this program as a cornerstone of department culture is a form of value signaling for residents, faculty, and prospective applicants, conveying to all that advancement of women in EM is part of our academic mission. It is notable that several participants provided a postsurvey free‐text response indicating positive impact on their sense of community within the department.

Future and ongoing directions for our women in EM program include expansion into our institution's medical school to address topics pertaining to gender and professional development at an earlier stage of training and beyond the specialty of EM, and expansion to an alumni network to provide ongoing professional support and mentorship.

## LIMITATIONS

We describe the inaugural year of a single‐site professional development program for women faculty and resident physicians in EM. Further data need to be collected on whether longitudinal participation across all training years of our program would alter the program's impact on participants. Quantifying concrete mentorship and sponsorship relationships, professional opportunities, and knowledge acquisition attributable to the program would be enlightening.

We were not able to capture the impact of the program on those who attended but who did not fill out a pre‐ or postprogram survey. Additionally, it would have been helpful to understand why some women in the department chose not to participate, whether due to lack of interest or lack of perceived need, or if attendance at a professional development program was less of an immediate priority for those who were already struggling or burned out, as previously hypothesized.^37^ We are also unable to capture the impact of independent use of the resources for those who did not attend sessions. We would also like to address in future studies why some reported less benefit than expected from taking part in the program—whether due to expectation mismatch, applicability of topics addressed, or an additional reason.

## CONCLUSIONS

We describe the conception and implementation of a novel structured curriculum to address the need for professional development and mentorship for women in academic emergency medicine. Success of the program was demonstrated in several ways, including meeting expectations for a majority of participants for impact on professional development, job satisfaction, professional well‐being, and personal well‐being; a positive impact on career choice and career trajectory for participants in each career stage; and participant engagement as measured by attendance to this voluntary program.

Recommendations for the successful implementation of similar programming to support professional development of women in academic medicine include early engagement of stakeholders, the application of data from a program‐specific needs assessment, early dissemination of session dates to allow for protected time off, and structured discussions with appropriate identification of presession resources. Continued identification of factors associated with successful professional development programs is critical to support the advancement of women in academic medicine.

## CONFLICT OF INTEREST

The authors have no potential conflicts to disclose.

## AUTHOR CONTRIBUTIONS

Simiao Li‐Sauerwine, Kimberly Bambach—study concept and design, acquisition of the data, analysis and interpretation of the data, drafting of the manuscript, critical revision of the manuscript for important intellectual content; Jillian McGrath, Jennifer Yee, Creagh T. Boulger, Jennifer Mitzman—study concept and design, analysis and interpretation of the data, drafting of the manuscript, critical revision of the manuscript for important intellectual content; Katherine M. Hunold—analysis and interpretation of the data, drafting of the manuscript, statistical expertise, critical revision of the manuscript for important intellectual content.

## Supporting information

Appendix S1

Appendix S2
