# Supplementary material for: Building a RAFFT: Impact of a professional development program for women faculty and residents in emergency medicine
Source: AEM Educ Train. 2022 Jun 23;6(3):e10763. doi: 10.1002/aet2.10763 (PMC9222109; doi:10.1002/aet2.10763)
Supplement: Supplementary file 1 — Appendix S1 [file AET2-6-e10763-s001.docx]

Appendix A: Pre-implementation survey

[Free text]

1. Do you think we need a Women in EM program? Why or why not?
2. What are your expectations or hopes for a Women in EM progam?

[5-point Likert scale from “not at all” to “a great deal”]

1. How much do you feel a Women in EM program will contribute to your professional development?
2. How much do you feel a Women in EM program will contribute to your personal wellness?
3. How much do you feel a Women in EM program will contribute to your professional identity?
4. How much do you feel a Women in EM program will contribute to your job satisfaction?

Current knowledge of the following areas

[5-point Likert scale from “none” to “I have a strong understanding”]

1. Imposter Syndrome
2. Professional Advancement - Salary and Contract Negotiation
3. Professional Advancement - Promotion
4. Professional Advancement - Advocating for Yourself
5. Professional Advancement - Goal Setting
6. Career Exploration & Job Specific Mentorship
7. Leadership Skills
8. Thriving Clinically - Nursing and Staff Communication
9. Thriving Clinically - Patient Communication
10. Feedback
11. Work Life Balance - Time Management
12. Work Life Balance - Relationships
13. Work Life Balance - Raising a Family
14. Mentorship
15. Supporting Each Other Professionally

Desire to learn more about the following areas

[5-point Likert scale from “not interested” to “very interested”]

1. Imposter Syndrome
2. Professional Advancement - Salary and Contract Negotiation
3. Professional Advancement - Promotion
4. Professional Advancement - Advocating for Yourself
5. Professional Advancement - Goal Setting
6. Career Exploration & Job Specific Mentorship
7. Leadership Skills
8. Thriving Clinically - Nursing and Staff Communication
9. Thriving Clinically - Patient Communication
10. Feedback
11. Work Life Balance - Time Management
12. Work Life Balance - Relationships
13. Work Life Balance - Raising a Family
14. Mentorship
15. Supporting Each Other Professionally

[Free text]

1. Additional suggestions for topics?
2. Other feedback?
