# Supplementary material for: Building a RAFFT: Impact of a professional development program for women faculty and residents in emergency medicine
Source: AEM Educ Train. 2022 Jun 23;6(3):e10763. doi: 10.1002/aet2.10763 (PMC9222109; doi:10.1002/aet2.10763)
Supplement: Supplementary file 2 — Appendix S2 [file AET2-6-e10763-s002.docx]

Appendix B: Post-implementation survey

[5-point Likert scale from “not at all” to “a great deal”]

1. How much do you feel a Women in EM program has contributed to your professional development?
2. How much do you feel a Women in EM program has contributed to your personal wellness?
3. How much do you feel a Women in EM program has contributed to your professional identity?
4. How much do you feel a Women in EM program has contributed to your job satisfaction?
5. To what extent has RAFFT influenced your CAREER CHOICE (e.g. community vs academics, desire to pursue leadership or mentorship roles)?
6. To what extent has RAFFT influenced your CAREER TRAJECTORY (e.g. decision to pursue chief resident role or faculty promotion)?

[Free text]

1. Do you think we need a Women in EM program (RAFFT)? Why or why not?
2. What do you feel like you have gained from our Women in EM program (RAFFT) in 2020-21?

Current knowledge of the following areas

[5-point Likert scale from “none” to “I have a strong understanding”]

1. Imposter Syndrome
2. Professional Advancement - Salary and Contract Negotiation
3. Professional Advancement - Promotion
4. Professional Advancement - Advocating for Yourself
5. Professional Advancement - Goal Setting
6. Career Exploration & Job Specific Mentorship
7. Leadership Skills
8. Thriving Clinically - Nursing and Staff Communication
9. Thriving Clinically - Patient Communication
10. Feedback
11. Work Life Balance - Time Management
12. Work Life Balance - Relationships
13. Work Life Balance - Raising a Family
14. Mentorship
15. Supporting Each Other Professionally

What RAFFT topic did you find most valuable to your professional development this year?

1. Supporting Each Other Professionally
2. Mentorship
3. Thriving Clinically - Nursing and Staff Communication
4. Advocating for Yourself
5. Career Exploration
6. Imposter Syndrome
7. Work Life Balance - Relationships
8. Work Life Balance - Raising a Family
9. Professional Advancement - Salary and Contract Negotiation
10. Pearls of Wisdom

[Free text]

1. Additional suggestions for topics?
2. Other feedback?
